# Benchmarking and refining probability-based models for nucleosome-DNA interaction

**DOI:** 10.1186/s12859-017-1569-0

**Published:** 2017-03-07

**Authors:** Marco Tompitak, Gerard T. Barkema, Helmut Schiessel

**Affiliations:** 1 Lorentz Institute, Leiden University, Niels Bohrweg 2, Leiden, 2333CA The Netherlands; 2 Institute for Theoretical Physics, Utrecht University, Princetonplein 5, Utrecht, 3584CC The Netherlands

**Keywords:** Sequence analysis, Nucleosome positioning, Modeling

## Abstract

**Background:**

In investigations of nucleosome positioning preferences, a model that assigns an affinity to a given sequence is necessary to make predictions. One important class of models, which treats a nucleosome sequence as a Markov chain, has been applied with success when informed with experimentally measured nucleosomal sequence preferences.

**Results:**

We find that we can also use such models as a fast approximative scheme for computationally expensive biophysical models, vastly increasing their reach. Employing these models in this way also allows us to benchmark them for the first time. Doing so for the approximative in silico models indirectly tells us about the accuracy we can expect of them when applied to real data.

**Conclusion:**

We find that models presented in the literature should perform well, but this performance depends on factors such as the order of the Markov model, the preprocessing of the probability distributions on which the model is based, and the size and quality of the sequence ensemble from which those distributions are calculated.

**Electronic supplementary material:**

The online version of this article (doi:10.1186/s12859-017-1569-0) contains supplementary material, which is available to authorized users.

## Background

It is well-established that nucleosomes have significant preferences as to DNA sequences they bind, and that these sequence preferences play an important role in a range of dynamic nucleosomal processes [1]. In order to better study correlations between sequence effects and biological function, it is necessary to get a grasp on the energetics of nucleosome-DNA interaction. Several approaches have been put forward. Sequence-dependent models that directly address the mechanics of DNA, such as the Rigid Base Pair Model [2] can be combined with a suitable model for the nucleosome to access the energetics of nucleosome-bound DNA [3–9].

Another option is to use a bioinformatics model that defines a probability distribution on the space of all possible nucleotide sequences. The logarithm of such a probability distribution relates linearly to the free energy of a sequence when wrapped into a nucleosome. One such probability-based model has been put forward by Segal et al. [10] and this particular model now proves to be of interest beyond its original purpose, in that it can also be used in silico to provide a computationally efficient approximation to biophysical models that are themselves computationally too intensive. By speeding up the calculation of the affinity of a sequence for the nucleosome by a factor of around 10^5^ (in an unoptimized implementation), this approximative scheme makes it possible to use the biophysical nucleosome model of Eslami-Mossallam et al. [5] to perform genome-wide analyses of nucleosome positioning signals. With this method, we have performed all-gene analyses of promoter regions for numerous organisms [11], a feat that would have been computationally intractable without it.

Here we perform an in-depth benchmarking analysis of this approximation to the Eslami-Mossallam et al. nucleosome model. We will examine to what accuracy the computationally efficient model approximates the predictions of the underlying model for the first chromosome of *S. cerevisiae*, and how this accuracy depends on several factors, such as the stringency of the assumptions that go into the approximation, the size of the sequence ensemble from which the model parameters are derived and the application of smoothing filters on those parameters. In doing so, we may also indirectly draw some conclusions as to the accuracy that may be expected of models such as that of Segal et al. [10], trained on experimental sequence ensembles.

## Methods

### Model

Since a nucleosome wraps 147 base pairs worth of DNA, the space of possible sequences contains 4^147^ or about 10^88^ possibilities. It is impossible to enumerate all of these, so a simple function is needed for the probability distribution.

Segal et al. do this by treating a DNA sequence as a Markov chain of order 1, where the probability of a nucleotide at a certain position depends only upon the preceding nucleotide. The probability of the sequence as a whole is the product of the probabilities of all the nucleotides it is composed of. More precisely, defining *S* as a sequence of length 147, consisting of nucleotides *S*_*i*_ with *i* from 1 to 147,

1 $$\begin{array}{*{20}l} P(S) &= P\left(\bigcap_{i=1}^{147} S_{i}\right) = P\left(S_{147}|\bigcap_{i=1}^{146} S_{i}\right) P\left(\bigcap_{i=1}^{146} S_{i}\right) \end{array} $$

2 $$\begin{array}{*{20}l} &= \prod_{n=1}^{147} P\left(S_{n}|\bigcap_{i=1}^{n-1} S_{i}\right),  \end{array} $$

where we have applied the chain rule of probabilities. If we now introduce the assumption we mentioned earlier, that the probability of a nucleotide depends only on the preceding nucleotide, we find the expression given by Segal et al., i.e.

3 $$\begin{array}{*{20}l} P(S) &= P(S_{1}) \prod_{n=2}^{147} P(S_{n}|S_{n-1}).  \end{array} $$

We should stress that the value of quantities like *P*(*S*_*n*_) depends not just on the value of *S*_*n*_ (i.e. which nucleotide is represented) but also on the position along the nucleosome, *n*. These probability distributions for, in the case of Segal et al., dinucleotides, can be obtained by analyzing a suitable ensemble of sequences that have high affinities for the nucleosome. Segal et al. generate such an ensemble from the genome they are interested in making predictions for, by mapping actual (in vitro) nucleosome positions along the DNA. Although the original model did not perform very well [12], this model has been applied with success – after a refinement of the model and employing a better training data set – to predicting nucleosome positions, by Field et al. [13] and Kaplan et al. [14].

These experimental probability distributions do not capture only the intrinsic mechanical preferences of the DNA. They also capture inherent biases in the sample (a genomic sequence necessarily contains only a small subset of all 10^88^ possible sequences of length 147) and biases of the experimental method. This makes it difficult to evaluate the accuracy of the model, since both the training of the model and its testing generally rely on the same experimental methods, and there is the risk that agreement between the model and reality is overestimated because the model correctly fits experimental artifacts. Therefore it becomes of interest to study the model in a theoretical framework, where we can isolate the purely mechanical effects.

Ensembles to inform this type of bioinformatics model can also be generated from a theoretical nucleosome model using the Mutation Monte Carlo (MMC) method [5]. This method adds mutation moves to a standard Monte Carlo simulation of a nucleosome, thereby sampling the Boltzmann probability distribution of pairs of sequences and spatial configurations (*S*,*θ*),

4 $$ P(S,\theta) = e^{-\beta E(S,\theta)}.  $$

By sampling the sequences during the MMC simulation, the spatial degrees of freedom of the nucleosome model are marginalized and one obtains the probability distribution of the sequences

5 $$ P(S) = \int d\theta e^{-\beta E(S,\theta)}  $$

and their free energy

6 $$ F(S) = -kT \log(P(S)).  $$

Note that in Eqs. 4–6 we have neglected the overall normalization of the probability distributions by the partition function *Z*, and hence a constant offset −*k* *T* log(*Z*) to the free energy. Because the probabilities we derive are simply relative frequencies with respect to our sequence ensemble, they are inherently normalized (i.e. summing them over all possible sequences gives unity) and we have no information on the partition function. This is not usually an impediment as we are mostly interested in relative energy differences.

Sampling the entire sequence space is not feasible, but making the same assumption about long-range correlations in the sequence preferences as Segal et al., we can assume that we may write our *P*(*S*) as in Eq. 3. It turns out it is feasible to produce a sequence ensemble large enough that the distributions *P*(*S*_*i*_|*S*_*i*−1_) may be determined.

### Generalization of the Dinucleotide Model

We used an MMC simulation of the model put forward by Eslami-Mossallam et al. at 1/6 of room temperature to generate an ensemble of 10^7^ sequences, from which the oligonucleotide distributions were derived (see Additional files 1, 2, and 3). At each position, we counted the number of instances of every mono-, di- and tri-nucleotide and divided these by the total number of sequences in order to obtain probability distributions.

This gives us the joint probability distribution *P*(*S*_*n*_∩*S*_*n*−1_) and not the conditional probability *P*(*S*_*n*_|*S*_*n*−1_) that we need for Eq. 3. This is easily remedied. We can rewrite Eq. 3 as

7 $$ P(S) = P(S_{1}) \prod_{n=2}^{147} \frac{P(S_{n} \cap S_{n-1})}{P(S_{n-1})} = \frac{\prod_{n=2}^{147} P(S_{n} \cap S_{n-1})}{\prod_{n=2}^{146}P(S_{n})}.  $$

We see that we can write this equation in terms of the probability distributions of mono- and dinucleotides that we can find from a sequence ensemble. Analogously, if we want to expand the model to trinucleotides, we insert the assumption that the probability of a nucleotide depends only on the previous two (creating a Markov chain of order two) and we find

8 $$ P(S) = \frac{\prod_{n=3}^{147} P(S_{n} \cap S_{n-1} \cap S_{n-2})}{\prod_{n=3}^{146} P(S_{n} \cap S_{n-1})}.  $$

This model can thus be applied using probability distributions for di- and trinucleotides, both to be obtained from a suitable sequence ensemble. The result easily generalizes to tetranucleotides and beyond. For mononucleotides, the model simplifies to

9 $$ P(S) = \prod_{i=1}^{147} P(S_{i}).  $$

### Analysis

Segal et al. test their model by predicting nucleosome positions along the genome they are studying and comparing with reality and they find that their model has some predictive power, even on genomes on which the method was not trained. However, their study is inevitably hampered by small statistics and their use of natural materials. The latter makes it difficult to judge the quality of their model.

The in silico methods allow us to test the model, as an approximation to the full underlying model, much more rigorously. Because we can explicitly calculate the energy of a given sequence, we can directly measure the correlation between the energy given by the theoretical nucleosome model and the probability calculated by the bioinformatics model. Using a standard Monte Carlo simulation of the nucleosome with a given sequence, we can measure the average energy

10 $$ \left<E\right>_{S} = \int d\theta E(S,\theta) e^{-\beta E(S,\theta)}  $$

of the sequence. Unfortunately, calculating the free energy using the Eslami-Mossallam nucleosome model is not straightforward, and we will be comparing <*E*>_*S*_ as predicted by the biophysical model with *F*(*S*) as predicted by the approximative model. At finite temperature, these quantities are not the same, differing by an entropic contribution. However, at low enough temperatures they converge. We will compare the predictions at 1/6th of room temperature, as some finite temperature is needed for the statistical simulations to function. In performing this comparison, we thus provide an upper limit for the discrepancy between the approximation and the real <*E*>_*S*_.

In order to generate an energy landscape with which to compare the results of the probability-based models, we take the first chromosome of *S. cerevisiae* (∼2×10^5^ base pairs) and perform a Monte Carlo simulation of the nucleosome wrapped with each 147-base-pair subsequence of the chromosome, using the nucleosome model put forth by Eslami-Mossallam et al. After letting the simulation equilibrate, we sample the energy of the system and take the average. In order to be able to compare this energy landscape with a probability landscape, we calculate the (Boltzmann) probability distribution and normalize this over the set of sequences for which we calculated the energy, and then take the logarithm to regain our (shifted) energy landscape.

Analogously, we use the probability-based model to generate a probability landscape of the same sequence. This we normalize over the set of sequences analyzed and convert to an energy using Eq. 6. We find that this procedure is about five orders of magnitude faster than using the full biophysical model.

We only know the free energy up to some constant offset, but by making sure both the real energy landscape given by the energetic model and the approximate energy landscape provided by our probability-based model have the same normalization, we can readily compare the two.

In doing so, we may draw some conclusions about this kind of Markov-chain model not only as it relates to the nucleosome model we consider here, but about the assumptions that go into it in general, i.e. the explicit assumption of short-range correlations and the implicit assumption that the sequence ensemble on which the model is being trained is large enough. To test the first assumption, we extend the dinucleotide model used by Segal et al. to mononucleotides (which assumes no correlations at all) and trinucleotides (which relaxes the assumption of short-range correlations) and compare their accuracy. For the second, we examine the accuracy of these three models as a function of the ensemble size on which they are trained.

## Results and discussion

**Fig. 1:**
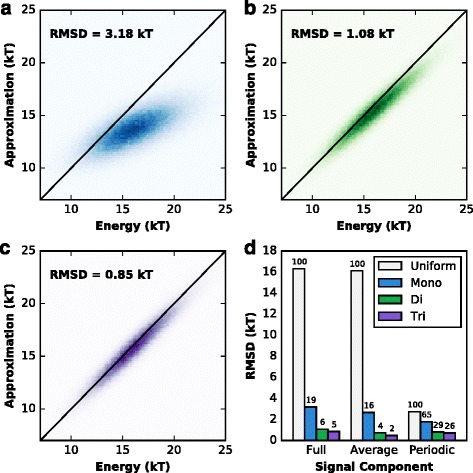
Accuracy analyses of the various models, benchmarked on the first chromosome of *S. cerevisiae*. **a** Histogram of the energy prediction pairs of the full model and mononucleotide approximative model for the same sequences. The black diagonal indicates perfect agreement. **b**, **c** As **a** for the dinucleotide and trinucleotide approximations, respectively. **d** Comparison of the root mean square deviations of the approximative predictions from those of the full model. The *grey bars* indicate the RMSDs of ‘bad’ models, defined for the Full and Average signals as a uniform landscape, and for the periodic signal as the real landscape shifted out of phase. The other values, for the mono-, di- and trinucleotide approximations are compared with these bad models. Indicated above each bar is a percentage indicating the value relative to the corresponding bad model

We tested and compared three different probability-based models, namely the Segal et al. dinucleotide model, its simplification to mononucleotides and its extension to trinucleotides. Following the methodology outlined in the previous section, we arrive at correlation plots for the energy as given by the energetic model and as predicted by the probability-based models. The results are presented in Fig. 1a–c.

As we might expect, the longer the oligonucleotides we use, the better the agreement becomes. An important cause of the deviation from perfect agreement, apart from the spread, is a clearly visible deviation in the slope. The mononucleotide model significantly underestimates the spread in energies. This means that the mononucleotide model is not capturing effects that set sequences apart from each other. This effect is expected and should be remedied by going to longer oligonucleotides. Indeed we see this deviation greatly decreased for the dinucleotide model, and even more so for the trinucleotide model.

For a more detailed grasp on the quality of the predictions, we separate out two components of the energy landscape that are important on their own. The first is the periodicity of the energy landscape. Due to the helical nature of DNA, energy landscapes for the nucleosome show a roughly 10-base-pair periodic signal. It is important that any model for nucleosome affinity gets the frequency and phase of this periodicity right. The second property, complementary to the periodicity, is the overall energy level of the sequence. This aspect will show us how well the model captures long-range effects.

For the purposes of benchmarking, we define the local average as the 11-base-pair running average of the energy landscape, i.e. over about one period. The pure periodicity of the signal we analyze by subtracting from the signal its local average as just defined, making the signal oscillate around zero. Our benchmarking results then consist of the root-mean-square deviation (RMSD) for the full signal (already presented in Fig. 1a–c), for the locally averaged signal and for the pure periodicity signal.

To get a sense of what the RMSD values we find actually mean, we compare them to the RMSD value we find when we use a bad model. For the overall signal and the locally averaged signal, we define this bad model to be one that contains no sequence information at all, i.e. a perfectly uniform landscape. For the periodicity, this is not such an interesting comparison because for a periodic signal, a uniform landscape is still right twice per period. Instead we utilize as a bad model the same signal, but shifted by half a period, to push it out of phase.

RMSD values gathered from such bad models tell us about the typical size of the structures in the energy landscape that our models need to predict. We can then measure the RMSD from our benchmarked models relative to this scale. Fig. 1d displays the results. We see a decrease in RMSD when going to longer oligonucleotides in each of the three cases. The dinucleotide model, as used by Segal et al., already performs well, with an overall RMSD of 7%. Noteworthy, it is much more accurate than the mononucleotide model. However, we see that we could improve our results still by going to trinucleotides. Especially the local average is predicted much more accurately by the trinucleotide model, cutting the RMSD by about a third.

### The Importance of Sample Size

Because we can produce large ensembles of sequences in silico with the Mutation Monte Carlo method, we are now also in a position to get a measure of how large an ensemble we need for our models to make accurate predictions.

In their 2006 study, Segal et al. manage to build an ensemble of ∼10^2^ sequences. Apart from the inherent biases that may be present in their ensemble due to their use of nonrandom yeast DNA, this is not a very large ensemble, and we should check what the effects of such limitations are.

In a later study, Kaplan et al. perform a similar study, where they obtain 35,000,000 sequence reads. [14] The ensemble is again trained on the yeast genome, which is some 12,000,000 base pairs long. The number 35,000,000 should therefore not be mistaken for the ensemble size. There must necessarily be many duplicate and strongly overlapping sequences in their ensemble, which arise artificially because only a small subset of sequence space is available for sampling. Giving a meaningful number for the effective sample size of such an ensemble is difficult. However, a sequence of ∼10^7^ base pairs can yield 10^4^−10^5^ completely non-overlapping nucleosome sequences, which we may employ as a conservative estimate.

Later similar work using the mouse [15] and human [16] genomes has yielded larger ensembles. These genomes are two orders of magnitude larger than that of yeast, and so also provide that many more non-overlapping sequences.

In our in silico simulations, we built an ensemble of 10^7^ independent sequences from which we derived our probability distributions. We took subsets of these sequences to see what the effects of smaller sample sizes are. The problem when statistics are small is not just that the probability distributions are less accurate. We additionally run into the issue that some rare dinucleotides simply do not appear in the ensemble at all. The estimate of their probability then becomes zero. The problem is that if any of the factors in Eq. 2 is zero, the entire product becomes zero, rendering the model useless.

For Segal et al. and Kaplan et al. this problem does not arise, because they do not need to work at low temperatures, but also because they apply a smoothing to their probability distributions. They estimate the probability *P*_*n*_(*S*_*n*_∩*S*_*n*_−1) of a dinucleotide by averaging over not just position *n*, but also *n*−1 and *n*+1. This is justified by the observation that their experimental method does not provide them with a sharp resolution down to the base-pair to begin with. The effect of such smoothing is not a priori clear, however. In a landscape with 10-bp periodicity, taking a 3-bp running average could have averse effects. Such smoothing may not be necessary or beneficial when applied to higher-resolution data.

We therefore propose an alternative method, where instead we consider a probability of zero, for any position, a failure of the ensemble. In such a case we conclude that we simply do not have any information, i.e. we artificially insert a flat conditional probability of 0.25.

**Fig. 2:**
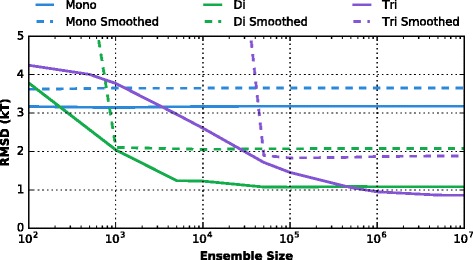
Variation of the RMSDs of the various models with the size of the sequence ensemble from which their parameters are calculated. *Solid lines:* zero-probability issues are dealt with by assuming zero information. *Dashed lines:* probability distributions are smoothed with a 3-bp running average. The performance when smoothing is strictly worse

In Fig. 2 are presented the RMSDs of the full landscape, as predicted by our probability-based models, with probability distributions derived from various ensemble sizes. We find that smoothing the distributions gives results that are strictly worse than simply assuming no information when an issue arises.

We can conclude from this plot that the model of Kaplan et al., even with a conservative estimate for their effective ensemble size, should perform well. The dinucleotide model converges to its maximum accuracy at only 10^4^ sequences. Of course, caveats surrounding the non-randomness of the DNA being sampled remain.

For larger experimental ensembles (e.g. [15] and [16]) it is advisable to move to a trinucleotide description. Starting from 5×10^5^ sequences, this model becomes more accurate than the dinucleotide model.

## Conclusions

With the methods available for the first time to produce sequence ensembles for nucleosome affinity based on an energetic model of the nucleosome, we investigated the capacity of a class of probability-based models to approximate real energetics. As an approximative scheme to the nucleosome model of Eslami-Mossallam et al. [5], we find errors on the order of 1 kT. This is not an insignificant disagreement, but depending on the application, this price may well be worth paying for the vast reduction in computational complexity by a factor of 10^5^ unoptimized. Vast increases in speed can also be expected for other complex biophysical models.

Considering the assumption of short-range correlations, we find that dinucleotide models such as those used by e.g. Field et al. and Kaplan et al. already perform well, with a root mean square deviation of about 2 *kT* (see Fig. 2). However, we also find that improvement could be achieved by going to a trinucleotide model (for large enough ensemble size), and by avoiding the smoothing of the probability distributions.

We also looked into the effects of small ensemble sizes, and we find that an ensemble such as used by Field et al., although caveats must be acknowledged as to likely inherent biases in their experiment, is sufficient for the dinucleotide model to reach its fundamental accuracy. For larger ensembles (10^6^ or more sequences) such as provided by the mouse or human genome, however, we recommend that the trinucleotide approximation be used for higher accuracy.

We hope, however, that our work will motivate the experimental community to look into mapping nucleosomal sequence preferences experimentally using more random DNA sequences than are provided by natural genomes. A starting point could be a very similar study done on DNA rings [17]. This would allow us to better examine the intrinsic sequence preferences of nucleosomes without biasing them towards a genomic context.

## Additional files

Additional file 1 Mononucleotide distributions. Table in tab-separated format denoting the mononucleotide probability distributions generated by our Mutation Monte Carlo simulation. (TSV 6 kb)

Additional file 2 Dinucleotide distributions. Table in tab-separated format denoting the dinucleotide probability distributions generated by our Mutation Monte Carlo simulation. (TSV 25 kb)

Additional file 3 Trinucleotide distributions Table in tab-separated format denoting the trinucleotide probability distributions generated by our Mutation Monte Carlo simulation. (TSV 106 kb)

## Abbreviations

MMC: Mutation Monte Carlo
RMSD: Root-Mean-Square Deviation
